# Establishment and evaluation of a PRRSV-sensitive porcine endometrial epithelial cell line by transfecting SV40 large T antigen

**DOI:** 10.1186/s12917-019-2051-1

**Published:** 2019-08-19

**Authors:** Kang Zhang, Houshen Li, Shasha Dong, Ying Liu, Dong Wang, Haichang Liu, Feng Su, Lijiang Ge, Yunliang Jiang

**Affiliations:** 1 Shandong Provincial Key Laboratory of Animal Biotechnology and Disease Control and Prevention, College of Animal Science and Veterinary Medicine, Shandong Agricultural University, 271018, No. 61 Daizong Street, Taian, Shandong China; 2 Department of Cardiology, Shandong First Medical University and Shandong Academy of Medical Science, Taian, 271000 China; 3 College of Chemistry and Material Science, Shandong Agricultural University, 271018, No. 61 Daizong Street, Taian, Shandong China; 4 Ningbo Defangyuan Biotech Co. Ltd., No.166 of Xinghaibei Road, County of Ninghai, Ningbo, Zhejiang China

**Keywords:** Porcine endometrial epithelial cell line, SV40T antigen, PRRSV-sensitivity, Lentrival vector, Replication efficiency

## Abstract

**Background:**

PRRSV is an infectious illness causing lung injury and abortion in sows. Cells apoptosis in the interface between the endometrium and fetal placenta is a crucial factor causing abortion. Previous study confirmed PRRSV could cause apoptosis of macrophages but rarely produced an obvious change in porcine endometrial epithelial cells (PECs). Recently, PRRSV-induced abortion was attributed to fetal placental and endometrium epithelial cells (Sn^+^ and CD163^+^) apoptosis. However, the mechanism of abortion is still unrevealed because of the limit of porcine endometrium epithelial cells (PEC). The aim of this study was to establish a stable immortalized PECs lines and use it to reveal the abortion mechanism.

**Results:**

In this study, highly purified primary PECs were harvested through differential digestion, and their characteristics were confirmed by CK18, ERɑ and PR staining. Cells were then immortalized by transfecting a lentiviral vector that expressed SV40 large T antigen. PECs lines were obtained after puromycin screening. Proliferation of cell line was evaluated by cell growth curve and cell cycle assays. Cell lines exhibited faster proliferation capacity than primary cells. Biological characteristics of cell line were assessed by Western blot, karyotype analysis and staining, which confirmed that the cell line retained the endometrium characteristics. Finally, PRRSV sensitivity was assessed; expression of Sn and CD163 indicated that primary PECs and cell lines were all potentially sensitive to PRRSV. PRRSV infection tests showed an obvious increase in apoptotic rate in the infected PEC cell line, which suggested its susceptibility.

**Conclusion:**

The newly constructed cell line is a useful tool for studying the mechanism of abortion caused by PRRSV.

## Background

Porcine reproductive and respiratory syndrome virus (PRRSV) is a single-stranded, positive-sense RNA virus with a small and enveloped RNA genome (approximately 15 kilobases) [1]. It is a member of the genus *Arterivirus*, family Arteriviridae, order Nidovirales. It can code for at least 14 non-structural proteins and eight structural proteins [2]. European genotype (type I) and the North American genotype (type II) are the two main genotypes of PRRSV [3, 4]. There are a variety of vaccines for controlling PRRS. However, its genetic variation, isolated from different places, increases the difficulty of developing vaccines against it. PRRS still remains one of the most important diseases causing massive economic loss to the swine industry worldwide [5]. PRRSV causes serious respiratory and reproductive disorders that are mainly attributed to cell susceptibility in these systems. PRRSV has been reported as infective in Marc-145 cells, PAM cells and endothelial cells in previous studies, of which monocyte/macrophages are the main target cells for PRRSV, especially in lungs, lymphoid tissues and placenta. Previous studies confirm that several mediators play important roles in these target cells [6, 7], of which Sn and CD163 are the most important receptors and have been widely investigated in previous studies [8, 9]. Sn (sialoadhesin) is a sialic acid adhesion transmembrane protein that acts as a moderator in preferential recognition of macrophages during external stimulus. Meanwhile CD163 belongs to class B of the cysteine-rich scavenger receptor superfamily (SRCRSF) [10], which mostly participate in anti-inflammatory processes.

PRRS is characterized by reproductive failure, such as abortion, producing weak piglets or mummified fetuses and respiratory problems in pigs. Previous studies demonstrate that PRRSV could result in placental cell apoptosis and lead to abortion in pregnant sows. Vital to placental function, endometrial epithelial cells form the interface between the endometrium and fetal placenta during gestation [11]. However, the susceptibility of endometrial epithelial cells to PRRSV is still unknown. The current research aimed to investigate the susceptibility of endometrial epithelial cells to PRRSV and to establish an infective porcine endometrial epithelial cell (PEC) line.

There are two common ways to establish a cell line: one is by transfecting a plasmid containing the HTERT (Human Telomerase Reverse Transcriptase) gene; another is to transfect vectors which can express the SV40 large T antigen (SV40T) gene. The former aims at initiating telomerase activation and preventing telomerase from shortening [12, 13]; the latter works by inducing tumorigenesis in cells [14]. SV40 is a primate polyomavirus discovered in 1960 [15]. Studies on SV40 focus mainly on basic viral and cellar functions including cell cycling and transcriptional control of gene expression [16]. SV40 shares a similar genomic structure to other polyomavirus, where a large non-coding region controls the expression of the early and late transcribed regions of the genome. Therefore, a large T antigen and a small T antigen protein could be created after alternate splicing. Of these two, large T antigen is a multi-functional protein responsible for dominating the host cells to produce conditions contributing to replication [17, 18]. Evidence shows that SV40T could induce tumorigenesis by targeting pRB and p53 both in a rodent model and in cultured cells [14]. More importantly, in practice, SV40T has a higher transfection and expression efficiency compared to HTERT.

This study first isolated and purified PRRSV-sensitive porcine endometrial epithelial cells and then established the cell line by transfer of SV40T gene.

## Results

### Isolated and purified primary PECs

**Fig. 1:**
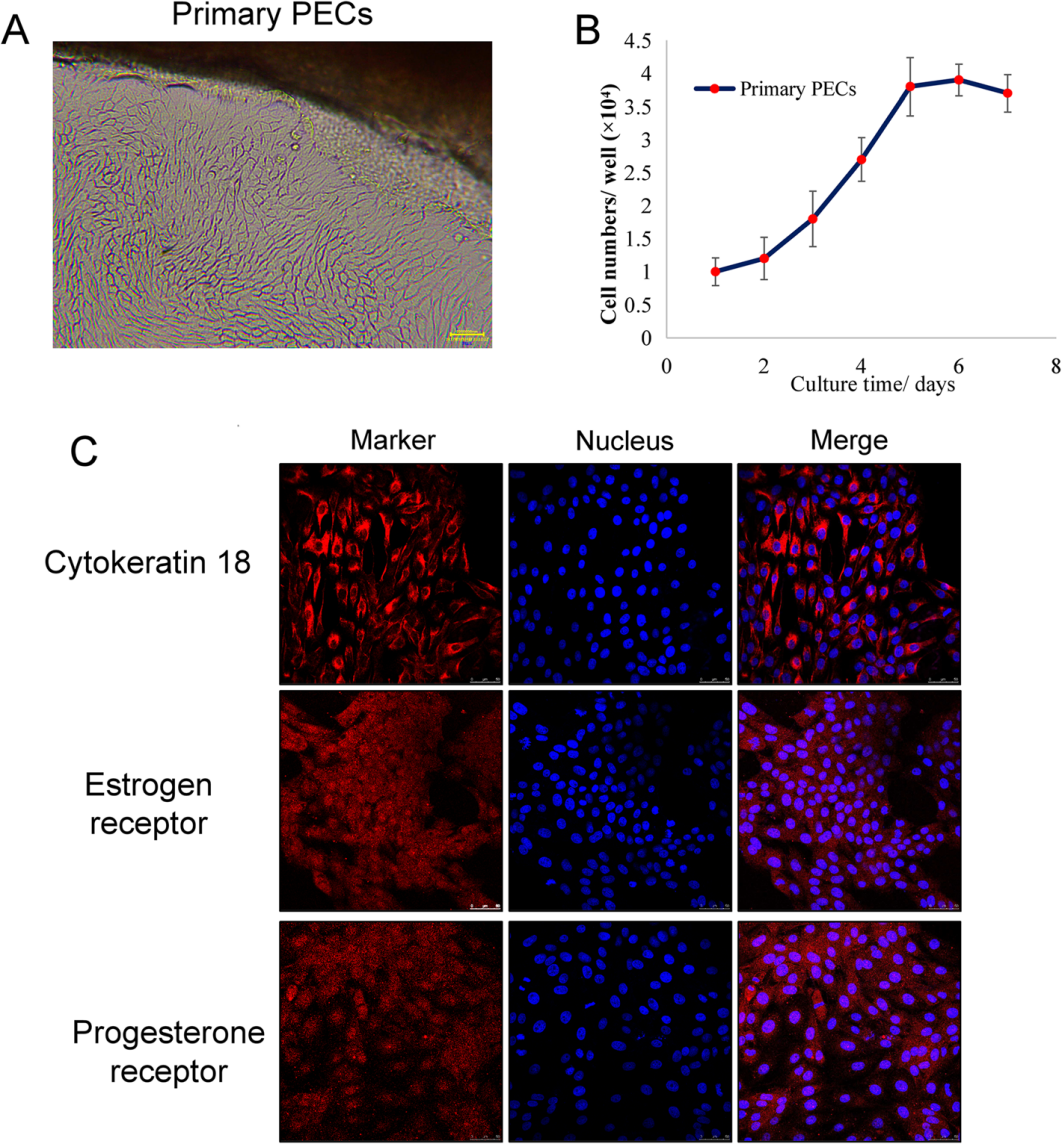
Characterization of primary porcine endometrial epithelial cells (PECs). **a** Primary PECs were observed after a 7 d culture under a light microscope (100×). **b** Primary PECs were cultured for 7 d and average cell numbers were counted on different days. **c** The immunofluorescence study for cytokeratin CK18, ERɑ and PR

After tissue block cell culture for 7 days, cells migrated from tissue block gradually and a monolayer of pure primary PECs was obtained, especially after differential attachment for 3 times. Furthermore, primary PECs and fibroblasts can easily be distinguished by morphology. In the current study, fibroblasts disappeared after differential digestion. Just like other epithelial cells, primary PECs grew in a monolayer and in the shape of paving stones (Fig. 1a).

The growth curve of primary PECs suggested a strong proliferative ability (Fig. 1b). Primary PECs were then identified using an immunofluorescence test. Cytokeratin-18 (CK-18), oestrogen receptor α (ERα) and progesterone receptor (PR), are the characteristic proteins in endometrial cells, and they were all positive when viewed under the microscope (Fig. 1c).

### Establishment of the PEC cell line

**Fig. 2:**
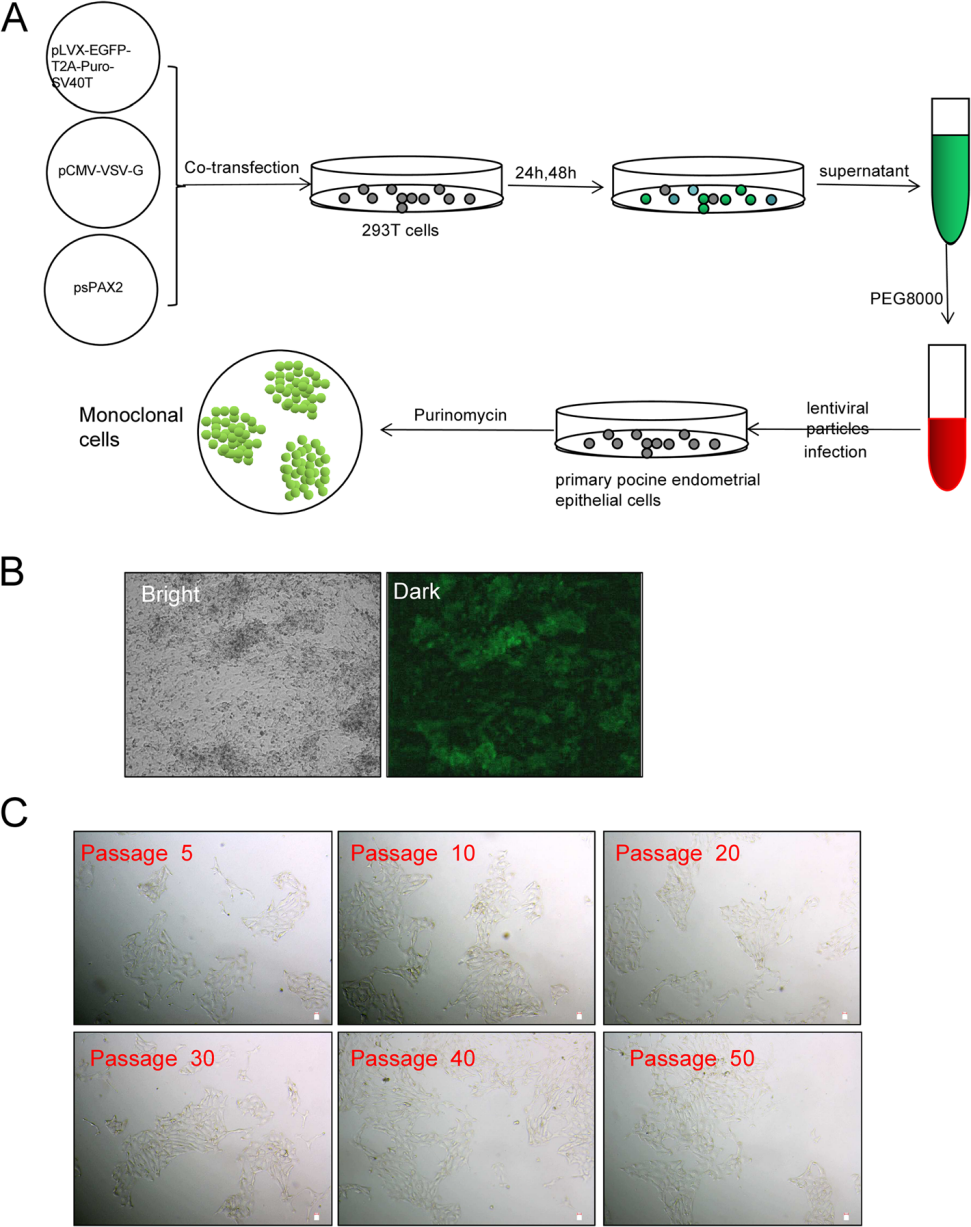
Process of SV40T transfection and obtaining a PEC cell line. **a** Lentiviral particles were packaged and used to infect primary PECs. After selection using puromycin, monoclonal cells were obtained. **b** Green fluorescence can be seen four days after infection of lentiviral particles. **c** Cellular morphology of the PEC cell line at 5, 10, 20, 30, 40 and 50 passages. No morphological differences were observed

The entire protocol used to build the cell line is shown in Fig. 2a. Purified high-titre lentiviral particles were obtained after co-transfection with pLVX-EGFP-T2A-Puro-SV40 vector, and pCMV-VSV-G and psMAX2 vectors in 293 T cells. The PEC cell line was screened with puromycin after infection with lentiviral particles in a 1:50 dilution solution (Fig. 2a).

Figure 2b shows the monoclonal puromycin-resistant cells after infection with lentiviral particles, the green fluorescence in the cells indicates the cells had a high purity (Fig. 2b). Cells were then obtained from a single clone and subsequent passage from one generation to another. Interestingly, green fluorescence of PECs lines was disappeared ultimately. The cells in Fig. 2c are cell line from different generations, all the cells exhibited paving stone characteristics and no obvious differences were found between primary cells with cell line (Fig. 2c).

### The PEC cell line sustained its biological characteristics

**Fig. 3:**
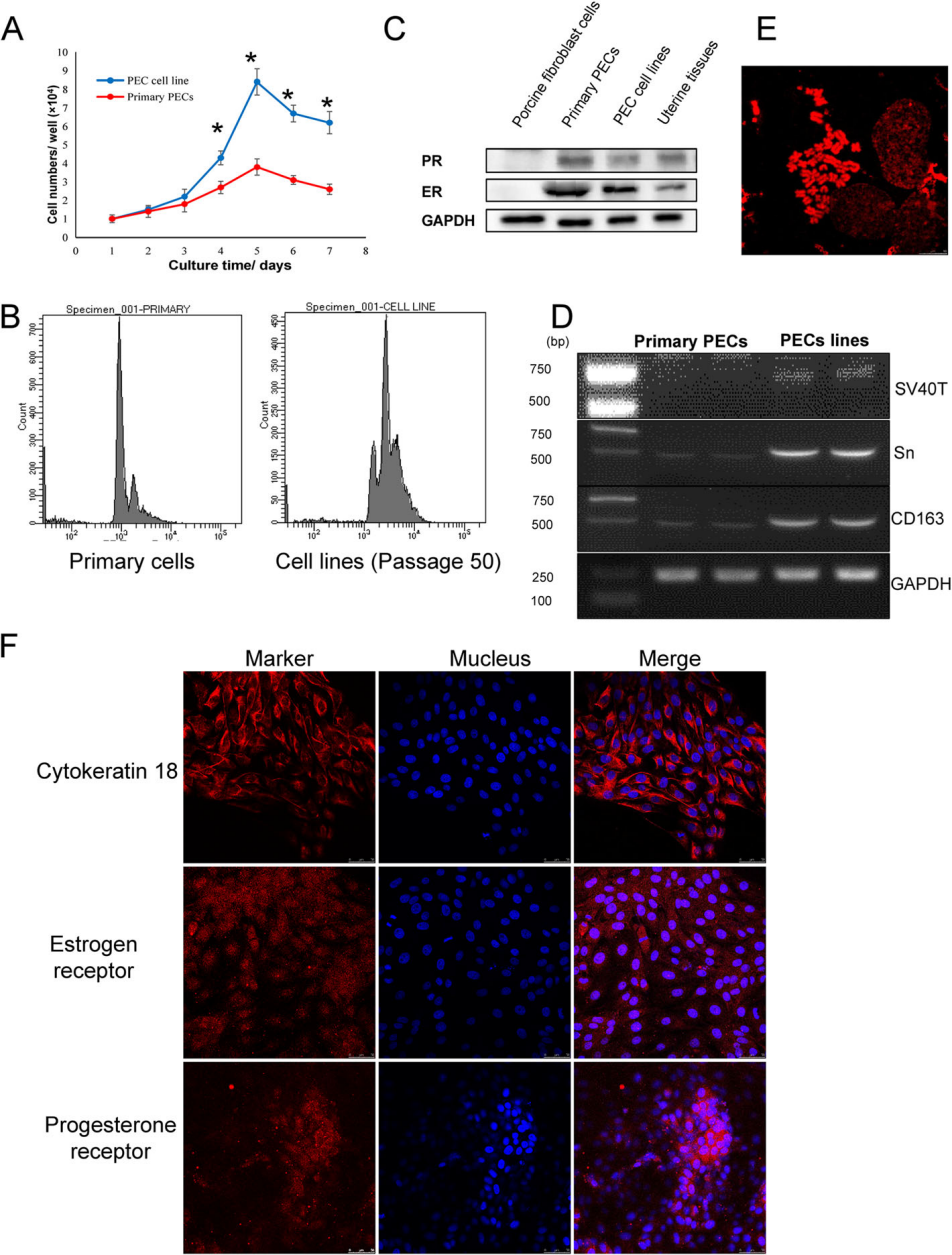
Characterization of the PEC cell line. **a** The comparison of proliferation between the PEC cell line and primary PECs. **b** Cell cycle comparison between primary PECs and the PEC cell line. (*:*p* < 0.05) All data were analysed using SPASS software. ANOVA was performed by one-way analysis. Values with *P* < 0.05 indicated a significant difference between the groups. **c** Western blot detection of ERɑ and PR. Expression of ERɑ and PR proteins are both shown in primary PECs and the PEC cell line. All data were analysed using SPASS software. ANOVA was performed by one-way analysis. Values with *P* < 0.05 indicated a significant difference between the groups. (*:*p* < 0.05) (D) RT-PCR detection of SV40T transcription. SV40T can be detected in the PEC cell line but not in primary PECs. **e** Karyotype analysis of the PEC cell line shows that the cells display a normal porcine karyotype. **f** The PEC cell line isolated was determined positive by immunofluorescence testing for cytokeratin CK18, ERɑ and PR, which confirmed its endometrial epithelial origin

Cell line proliferation characteristics were evaluated from its growth curve and its cell cycle. Compared with primary PECs, cell line (50 passage) proliferation was significantly faster (*P* < 0.05) (Fig. 3a). Subsequently, the cell cycle tests showed that the PEC cell line had longer G0 and S phases compared to those of primary PECs (*p* < 0.05) (Fig. 3b).

Biological attributes of the PEC cell line were then evaluated using its symbolic marker. Western blot tests showed that the cell line reserved its PR and ERα receptors as primary PECs (Fig. 3c). RT-PCR tests indicated the cells sustained CD163 and Sn properties (Fig. 3d). However, SV40T transcription only happened in the PEC cell line and not in primary cells (Fig. 3d).

Karyotype analysis in the cell line was used to locate chromosome loss. No abnormal chromosomes were found according to karyotype analysis, and the PEC cell line had a normal diploid chromosome number (2n = 38; Fig. 3e).

Endometrial epithelial cell characteristics were evaluated using immunofluorescence assays. Expression of CK-18, ERα and PR exhibit red fluorescence in Fig. 3f.

### Sensitivity to PRRSV

**Fig. 4:**
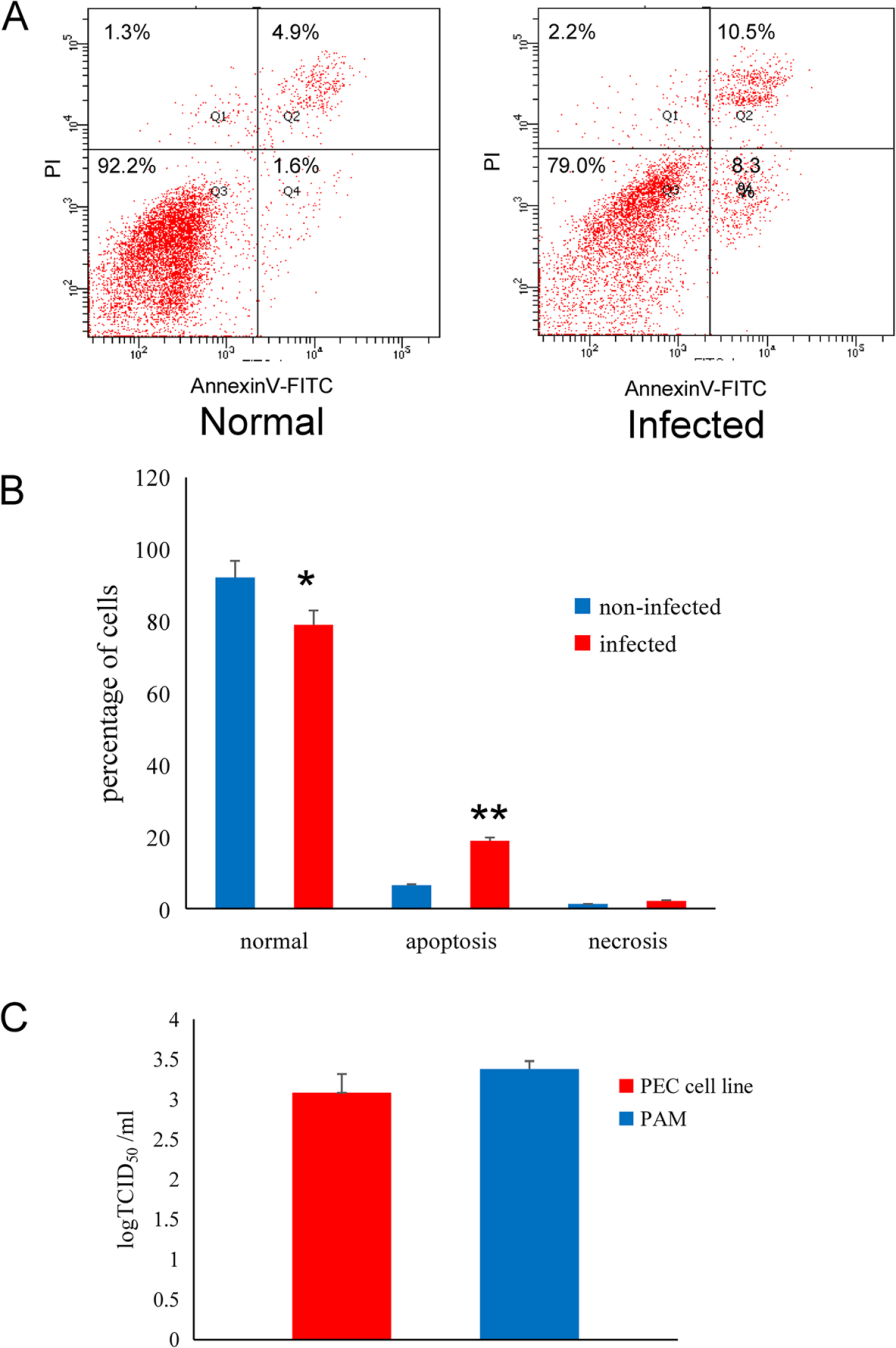
Susceptibility of PRRSV in the PEC cell line. **a** Comparison of apoptosis between normal and PRRSV-infected PEC cell lines. **b** A significant difference was observed between the normal and apoptotic cells after infected (* *p* < 0.05), the data were presented as Mean ± SEM and analysed by one-way analysis. **c** Comparison of PRRSV replication efficiency in the PEC cell line and PAMs (* *p >* 0.05), the data were presented as Mean ± SEM and analysed by one-way analyse

PRRSV-sensitivity of the PEC cell line was evaluated by its apoptosis ratio; the PRRSV-infected PECs apoptosis ratio was significantly higher than the non-infected one (Fig. 4a, b). Subsequently, virus replication was evaluated by its TCID_50_; both PAMs and the PEC cell line were infected with PRRSV successfully for 48 h, and supernatants collected then were used to infect PAMs for virus titration. The viral titre in the PEC cell line was 3.21 logTCID_50_ and which was slightly lower than that in the PAMs (3.45 logTCID_50_) (*p* > 0.05), (the data were presented as Mean ± SEM and analyzed by one-way analysis).

## Discussion

It is known that monocytes/macrophages are the main host cells for PRRSV infection because all these cells, such as MA-104, CL2621, MARC-145, PAMs and blood monocytes, are susceptible to PRRSV. However, monocytes are obtained from pigs and all other cells originate from kidney tissue of African green monkeys. So it is questionable whether these monkey cells correlate with porcine cells [19]. Previous studies have shown that the apoptosis of endometrial epithelial cells is closely linked to the termination of gestation [20]; however, only a few studies concentrate on the underlying mechanism. When infected with PRRSV, PAMs will die due to apoptosis [20]. Therefore, if apoptosis can be induced in the PRRSV-infected endometrial epithelial cells, it can then be confirmed that endometrial epithelial cells are also the target of PRRSV and can be used for further research on PRRSV related abortion in pigs.

In another study, Feng et al. isolated primary PECs by digesting endometrial tissue pieces with collagenase at 37 °C overnight [21]. They purified primary PECs as fibroblasts attached themselves to the dish surface within 30 min, but this process takes at least 2 h for PECs. In the present study, primary PECs were harvested using tissue blocks after being cultured. Cells migrated out of blocks after cultured for four days. Most of those were epithelial cells and mixed with a small quantity of fibroblasts on attaching monolayer. High purity primary PECs can be harvested since fibroblasts can be removed by differential digestion. Meanwhile, other stromal cells are still unable to survive due to inappropriate culture conditions. CK18 is a cell-specific protein marker expressed mainly in epithelial cells coupled to multiple intracellular signaling pathways [22]. Almost all the cells exhibited fluorescence, indicating that cells isolated from the endometrium were epithelial cells and of high purity (> 98%) that met with experimental requirements. In the present study, some functional proteins in the endometrial epithelial cells, such as ERɑ and PR [22, 23] were also detected. The above evidence indicated that these monolayer cells with functional proteins are endometrial epithelial cells.

Previous studies have demonstrated that cells become immortalized provided they can bypass senescence and any crises [24, 25]. SV40 large T antigen binds to and inactivates tumour suppressor proteins such as p53 and pRB [26–28]. Expression of this oncoprotein promotes cells to bypass senescence and retain their ability to proliferate. In the present study, SV40 large T antigen expression vectors were transfected into primary PECs using packaged lentiviral particles for the immortalization of cells. After screening with puromycin, the cells originating from a single cell clone showed a notably faster growth rate and a higher adherence efficiency as compared to primary PECs. The PEC cell line underwent passages for over 50 generations while maintaining a homogeneous population and invariant properties. Additionally, the characteristic proteins found in primary PECs e.g., CK18, ERɑ and PR could also be detected in the PEC cell line. In addition, the expression of ERɑ and PR were also quantified by western blotting. Results showed that there was no reduction in the expression of the two proteins in PECs compared to primary PECs. Therefore, the properties of the PECs reconstituted by transfection were similar to those of primary PECs. Many cell lines transfected with SV40T have numerous karyotypic abnormalities, such as Chromosome doubling [29]. However, karyotype analysis in our current study indicated that the PEC cell line had normal chromosomal numbers.

It was expected that the cell line would be sensitive to PRRSV. Sn is the macrophage-specific cell adhesion molecule expressed predominantly on the surface of macrophages. It mediates PRRSV endocytosis by binding to sialic acids on the PRRSV virion. Additionally, PRRSV can also enter cells by exploiting CD163 [30]. Through over-expression of CD163, many PRRSV-nonsusceptible cell lines, such as BHK-21, PK-0809 and NLFK, become fully susceptible to PRRSV and can produce high viral titres [6]. CD163 is likely one of the most essential receptor for PRRSV [8]. As important PRRSV receptors, Sn and CD163 can be amplified in primary PECs and the PEC cell line, indicating that endometrial epithelial cells are also susceptible to PRRSV. Interestingly, Feng et al. (2013) found that CD163 can be transcribed in PAMs but not in the cell line (PEE) they generate. However, in our experiment, transcripts of CD163 could be detected in both the PEC cell line and primary PECs. Previous studies have demonstrated that apoptosis is one of the most important mechanism for PRRSV-induced reproductive failure. In our research, we noted that the apoptotic rate was significantly increased after the PEC cell line was infected with PRRSV.

Another important step was undertaken to confirm that the PEC cell line can be used for PRRSV multiplication or to produce high titres of the infectious virus. Surprisingly, our research demonstrated that PRRSV-infected cells showed a cytopathic effect after 24 h and this became more obvious after 48 h. Supernatants from PAMs and PECs that were infected with PRRSV were used for viral titres. PAMs are a common cell type that are most sensitive to PRRSV and provide a suitable place for viral replication. In the present research, though PAMs were more successful in producing high titres of virus compared to the PEC cell line, we found that the PEC cell line can also be used for viral reproduction. Thereby, it is inferred that PEC cell lines can be used in future research associated with PRRSV-induced endometrial epithelial cell apoptosis. Although the underlying mechanism for apoptosis in PRRSV-infected endometrial epithelial cells is still unclear, the expression of Sn and CD163 maybe one of the reasons for obvious changes in apoptosis. Sensitivity of the PEC cell line to PRRSV provides greater insight into our understanding of the stable PRRSV-colonizing cell lines and future development of a PRRSV vaccine.

When transfected into cells, the *SV40T* and *EGFP* genes can be efficiently integrated into the genome by lentiviral particles [31]. However, this integration is random. In the current study, interestingly, green fluorescence became gradually weaker and then disappeared when the cell line had been passaged over 50 generations. There are studies confirming that exogenous genes can be silenced due to methylation which occurs in the CMV promoter region [32, 33] . With the processing of culturing, the expression of exogenous genes decreases gradually because methylation occurring in the CMV promoter region increased gradually [34].

## Conclusion

In summary, the establishment of a PEC cell line in the current study could be useful for further research on the mechanism of PRRSV infection in endometrial epithelial cells; in addition, PECs can be used for the growth of PRRSV.

## Methods

### Primary PEC culture

All sows used in this study were housed in appropriate livestock housing and fed ad libitum. Sows were bought from ZhengDa company (Taian, Shandong, China) and sacrificed by an injection of sodium barbital (5 mg/kg) after anesthesia (subcutaneous injection). Endometrial tissue was collected from non-vaccinated adult sows (Chinese local white breed). Uterine cavity was cut off by longitudinal line and endometrial tissue was separated. Tissues were washed with PBS thrice, and then minced into several pieces of around 1 mm^3^. Tissue blocks were placed into 60 mm petri dishes with DMEM/F12 (containing 10% FBS and 10 ng/mL EGF) in cell incubator at 37 °C containing 5% CO_2_. The medium was refreshed every two days.

### Virus package, cell transfection and single clone selection

Human 293 T cells were purchased from the cell bank of Chinese academy of sciences and was cultured in 100-mm petri dishes. Lentiviral package vectors psPAX2 (6 μg, Addgene, #12260), pCMV-VSV-G (6 μg, Addgene, #8454) and pLVX-EGFP-T2A-Puro-SV40T (7.5 μg) were co-transfected into human 293 T cells to produce lentiviral particles. Medium was collected at 48 h and 72 h, and filtered with a 0.45 μm filter (Millex®-HV). The viral supernatants were mixed with 60% 5 × PEG8000 at 4 °Covernight, and centrifuged at 4000 *g* for 45 min. After removing supernatants, sedimentary lentiviral particles were resuspended in DMEM medium. Lentivirals (containing 5 μg/ml polybrene) were used to infect primary PECs for 24 h, and then replaced with fresh medium. Four days later, these primary PECs were selected in fresh medium containing puromycin (1 μg/ml) for three weeks. After selection, several puromycin-resistant cell clones were chosen for subsequent cell culture. Cells were digested and collected from a single clone, then cultured into 6-well plates in cell incubator at 37 °C containing 5% CO_2_. Subsequently, the supernatant was placed into another new plate after 10mins sedimentation. The epithelial cells were existed into the new plate.

### Immunofluorescence assay

The PEC cell line and primary PECs were seeded on microslides. Cells were fixed with 4% paraformaldehyde for 1 h at room temperature after culturing 48 h. Triton X-100 (0.5%) was used to permeabilize cells for 10 min. Cells were washed with PBS thrice for 5 min each and then blocked with 10% FBS for 1 h. Finally, anti-CK18 (cytokeratin 18), ER2α (estrogen receptor α) or PR (progesterone receptor) polyclonal antibodies at a dilution of 1:100 were added, respectively, and incubated at 4 °C overnight. After a further wash in PBS, cells were mixed with diluted Alexa Fluor 555-Labeled Donkey Anti-Rabbit IgG (Beyotime) (1:100) and protected from light at 37 °C for 1 h. After adding appropriate Hoechst 33342 (Beyotime) for 10 min and washing with PBS, these fluorescent positive cells were visualized under a confocal microscope.

### RNA extraction and RT-PCR

The PEC cell line and primary PECs were seeded on 6-well plates for 48 h. Total RNA was extracted utilizing TRIzol reagent (Invitrogen) according to the manufacturer’s protocol. A PrimeScript™ RT reagent kit with gDNA Eraser (Perfect Real Time, TaKaRa) was used to convert RNA to cDNA. Real-time PCR was performed using the 2 × EasyTaq PCR SuperMix (Transgen Biotech) with a Biorad PCR detection system. PCR was performed with the following program: 1 cycle of denaturation at 94 °C for 300 s; 34 cycles of denaturation at 94 °C for 30 s, annealing at 60 °C for 30 s and at 72 °C for a further 30 s; followed by 1 cycle at 72 °C for 300 s. The primer pairs used for detection are listed in Table 1.

**Table 1:** Primers used in this study

Gene name	Primer pair	Sequence (5′ → 3′)	Length (bp)
SV40T	Forward primer	AGTGGCTGGGCTGTTCTTTT	671
	Reverse primer	ATGGGAGCAGTGGTGGAATG	
Sn	Forward primer	GGATTCGGGCTTCTACTTCTG	489
	Reverse primer	TACCAGGAAAAACGGGTGTC	
CD163	Forward primer	TGTGGAAGTGCTGTCAGTTTCT	495
	Reverse primer	AAATGTGTCCAGTTCCCTCACT	
GAPDH	Forward primer	TGGTGAAGGTCGGAGTGAAC	225
	Reverse primer	GGAAGATGGTGATGGGATTTC	

### Western blotting

Both primary PECs and the PEC cell line were collected using 0.25% trypsin, and then centrifuged at 800 *g* to remove trypsin. After two washes with PBS, these cells were resuspended with RIPA buffer containing PMSF (1 mM) complete protease inhibitors. Cells were lysed in an ice-bath for 30 min and centrifuged at 10500 *g* for 10 min to collect the supernatant. The concentration of total protein was determined using a BCA Protein Assay Kit. Proteins were separated by SDS-PAGE and transferred to PVDF membranes. Membranes were blocked with 5% skim milk powder for an hour and incubated with anti-E2α (1:1000, Abcam), anti-PR (1:1000, Abcam) and anti-GAPDH (1:10000, Abcam) polyclonal antibodies overnight at 4 °C. After three washes with TBST, the membrane was incubated with specific goat anti-rabbit IgG (H&L) secondary antibodies for 1 h at room temperature, and then washed with TBST thrice. Finally, it was covered with BeyoECL Moon ECL Kit (Beyotime), and its chemiluminescence was detected using Chemiluminescent Detector.

### Cell-cycle and growth curve

Primary PECs and the PEC cell line were collected and washed with cold PBS twice, and then resuspended with cold 75% ethyl alcohol and fixed at 4 °C overnight. The first centrifugation was at 800 *g* for 5 min followed by washing with cold PBS twice to remove the 75% ethyl alcohol. The second centrifugation was implemented to remove the supernatant, then 500 μl of PBS (containing PI, 50 μg/mL) was added. Finally, the above cells were detected using a BD FACSCalibur™ flow cytometer.

Primary PECs and the PEC cell line were seeded on a 96-well plate (the number of cells was 1000/well) for 7 days. Cell number was counted each day.

### Karyotype analysis

The PEC cell line was treated with colchicine (0.1 μg/ml) for 4 h until many single-layer cells contracted their bulge and vacated a void. After centrifugation, the supernatant was removed and the cells were placed in 5 mL 0.075 mol/L KCl and incubated at 37 °C for 20 min. Subsequent centrifugation removed the supernatant. Cells were then fixed with 1 ml fixation solution (methyl alcohol:glacial acetic acid = 3:1) and left for 5 min; they were then centrifuged to remove the supernatant. An aliquot of 3 mL of fixation solution was then added to cells for 30 min. Cells were centrifuged and fixed again. A further centrifugation removed the supernatant. Then, 1 mL of fixation solution was added (containing PI, 10 μg/mL). Finally, the cell suspension was pipetted onto glass slides (dipped in 95% ethyl alcohol, stored at 4 °C, and then mounted) and the number of chromosomes was counted using confocal microscopy.

### Cell apoptosis after infection with PRRSV

PEC cell line cells were collected after infection with PRRSV for 48 h. Cellular apoptosis was detected using a BD Pharmingen™ FITC Annexin V Apoptosis Detection Kit according to the manufacturer’s protocol.

### Replication efficiency of PRRSV in the PEC cell line

PEC cell line cells and porcine alveolar macrophages (PAMs) (PAMs were purchased from ATCC company and kept in storing in the lab) were both infected with PRRSV for 48 h. After infection, cells were given normal fresh medium for 24 h and then supernatants were collected respectively. The supernatant was used for virus titration.

### Statistical analysis

All data were analysed using SPASS software. ANOVA was performed by one-way analysis. Values with *P* < 0.05 indicated a significant difference between the groups.

## Data Availability

Professor Feng Su and Yunliang Jiang can be contacted if someone wants to request the data.

## Abbreviations

CK-18: Cytokeratin-18
ERα: Oestrogen receptor α
PAM: Porcine alveolar macrophages
PR: Progesterone receptor
PRRSV: Porcine reproductive and respiratory syndrome virus
Sn: Sialoadhesin
SV40T: SV40 large T antigen
